# Comparative analysis of neuroinflammatory pathways in Alzheimer’s disease, Parkinson’s disease, and multiple sclerosis: insights into similarities and distinctions

**DOI:** 10.3389/fnins.2025.1579511

**Published:** 2025-04-29

**Authors:** Julia Doroszkiewicz, Izabela Winkel, Barbara Mroczko

**Affiliations:** ^1^Department of Neurodegeneration Diagnostics, Medical University of Bialystok, Bialystok, Poland; ^2^Dementia Disorders Centre, Medical University of Wroclaw, Scinawa, Poland; ^3^Department of Biochemical Diagnostics, Medical University of Bialystok, Bialystok, Poland

**Keywords:** neurodegeneration, neuroinflammation, Alzheimer’s disease, Parkinson’s disease, multiple Sclerosis

## Abstract

Neurodegenerative diseases, contributing to the significant socioeconomic burden due to aging society, are gaining increasing interest. Despite each disease having different etiologies, neuroinflammation is believed to play a crucial role in Alzheimer’s disease (AD), Parkinson’s disease (PD), and multiple sclerosis (MS). In addition to the pathogenic function of inflammation in the brain there is growing evidence that immune responses are essential for neuroregeneration. This review compares and contrasts the neuroinflammatory pathways that selected neurodegenerative diseases share and have in common. In AD, tau tangles and beta-amyloid plaques cause microglia and astrocytes to become activated in an inflammatory response. Alpha-synuclein aggregation stimulate neuroinflammation in Parkinson’s disease, especially in the substantia nigra. In Multiple Sclerosis an autoimmune attack on myelin is connected to inflammation via invading immune cells. Commonalities include the release of pro-inflammatory mediators like cytokines and activation of signaling pathways such as NF-κB and MAPK. Comprehending these common routes is essential for discovering early diagnostic possibilities for the diseases and possible tailored treatments. Our work underscores the potential for insights into disease mechanisms. Identifying common targets offers promise for advancing our understanding and potential future treatment approaches across these debilitating disorders.

## 1 Introduction

Neurodegeneration is the term used to describe pathological states that mainly damages neurons. This process is irreversible, with a progressive loss of structure and function of the neurons. In clinical practice, neurodegenerative diseases include a broad category of neurological ailments. These conditions include a range of clinical and pathological features and affect different populations of neurons in certain regions of the central nervous system (CNS). Classical neurodegenerative diseases include Parkinson’s disease (PD) and Alzheimer’s disease (AD) (Breijyeh and Karaman, 2020; Marogianni et al., 2020). Furthermore, multiple sclerosis (MS), which is mainly neuroinflammatory disease, can be regarded as a neurodegenerative illness (Garton et al., 2024). Although having distinct pathogenetic processes, all of these disorders share the characteristic of persistent neuroinflammation. The aging population, where neurodegenerative diseases are becoming common cause of impairment, has drawn more focus to the pathogenic mechanisms underlying neurodegenerative diseases. Immune responses play a major role in dementia. As a result, immune related genetic abnormalities have been proposed as risk factors for neurodegeneration. Ischemia, neurodegenerative diseases, immune mediated disorders, infections, and trauma are all known to cause immunological activation inside the CNS, which often has the potential to aggravate neural damage. Delayed regeneration happens when microglia’s ability to remove debris from myelin injury is hampered. As more and more underlying molecular pathways become clear, there is strong evidence to support the development of treatment approaches that might control neuroinflammation in order to avert CNS diseases. The link between neuroinflammation and the pathophysiology of neurodegenerative illnesses as well as the critical inflammatory signaling pathways implicated in neurodegeneration are the main topics discussed in this review.

## 2 Neuroinflammation

### 2.1 Innate responses in CNS

It is known that the CNS experiences both innate and adaptive inflammatory responses. An essential first line of defense for opsonizing and eliminating apoptotic cells is the activation of the innate immune system. Additionally, by secreting different cytokines and chemokines that cause adhesion molecules on the BBB (Blood Brain Barrier) and costimulatory molecule expression on microglia, innate immune responses attract cells of the adaptive immune system (Amor et al., 2010). Innate immunity serves as the body’s initial line of defense against infections and is also essential for tissue healing, removing apoptotic cells and other biological detritus, as well as fighting off tumors. Macrophages, natural killer (NK) cells, mast cells, oligodendrocytes, and neurons help in the responses to the microglia and astrocytes which are the primary innate immune cells in the CNS. These are examples of pathogen-associated molecular patterns (PAMPs) and damage-associated molecular patterns (DAMPs) (Lill et al., 2015). Cellular receptors that identify PAMPS and DAMPs, like endogenous molecules, heat-shock proteins (HSPs), viral and bacterial antigens, as well as oxidized lipids, are known as nuclear oligomerization domain-like receptors (NLRs), C-type lectins, oxidized lipoprotein detectors, and Toll-like receptors (TLR). NLRs are also important players in the inflammasome. The relationship between AD and PD and an uncommon mutation in TREM2 may be explained by the fact that innate receptors, such as triggering receptor expressed on myeloid cells 2 (TREM2), are essential for assisting in the removal of dying cells, myelin debris, and aggregated proteins (Stephenson et al., 2018).

The primary resident innate immune cells in the central nervous system, microglia, perform a variety of roles. One of them is that they modify brain circuits during development by removing waste products and controlling cell death in response to inflammation or CNS damage. Based on the production of chemokines and cytokines *in vivo*, microglia that have undergone differential activation are frequently categorized as either classical, described as pro-inflammatory (M1) or alternative, rather anti-inflammatory (M2) (Sica and Mantovani, 2012; Mirarchi et al., 2024). The application of parabiosis in mice has demonstrated how aging affects the switching between these polarizations, which is essential for remyelination (Ruckh et al., 2012). Microglia secrete pro-inflammatory as well as anti-inflammatory molecules, which can either be useful or deleterious in neurodegenerative diseases (Wes et al., 2016), for example, microglia removal in a mouse model of AD reduced neuronal loss without changing Aβ pathogenesis (Spangenberg et al., 2016). Different transcriptome profiles of microglia can be differentiated by the microbiota, aging, neuropathological conditions, and localization in CNS (Erny et al., 2015; Grabert et al., 2016; Wes et al., 2016). In recent years, a unique subset of microglia known as disease-associated microglia (DAM), a fraction of microglia with a different transcriptional and functional signature, was discovered in immune cells of the CNS of neurodegenerative disorders (Keren-Shaul et al., 2017; Wu and Eisel, 2023). DAM is molecularly identified as immune cells that display the typical microglial markers Iba1, Cst3, and Hexb, as well as the upregulation of “neurodegeneration” genes, including numerous recognized AD risk genes (e.g., Apoe, Lpl, Trem2, and Ctsd), and the downregulation of “homeostatic” gene set (e.g., P2ry12/P2ry13, Cx3cr1, Cst3 and Tmem119) (Hansen et al., 2018; Wang, 2021; Wu and Eisel, 2023).

Astrocytes are specialized glial cells, primarily existing as protoplasmic variants in grey matter and fibrous variants in white matter, making them the most prevalent type of glial cells in the brain (Colombo and Farina, 2016). Astrocytes were once thought to be purely inactive, but more recent research has shown that they are vital and actively involved in maintaining brain homeostasis (Oksanen et al., 2019). They govern the production of neurotrophin, regulate blood flow, preserve the blood–brain barrier, supply energy metabolites to neurons, adjust synaptic activity, eliminate dead cells, and regulate the extracellular balance of ions, fluid, and transmitters as well as the creation of scars (Sofroniew, 2009; Colombo and Farina, 2016; Oksanen et al., 2019). Subpopulations of astrocytes have been shown to produce pro-inflammatory mediators and immunoregulatory mediators, which are similar to the M1/M2 polarization of macrophages and microglia in causing the different types of activation (A1 or A2). Considered neuroinflammatory, the A1 astrocytes that are induced by Il-1a, TNFα, and C1q, release a neurotoxin that induces rapid death of neurons and oligodendrocytes and *in vitro* studies demonstrated their ability to harm neurons, they also induce apoptosis and inhibit T helper cell activation, proliferation, and function of activated T-cells. A2 astrocytes, on the other hand, support synapse repair, neuronal development, and survival (Liddelow and Barres, 2017). Numerous factors can cause astrocytes to react, and they are commonly seen to be hypertrophic in a variety of neurodegenerative diseases, such as amyotrophic lateral sclerosis (ALS), multiple sclerosis (MS), stroke, TBI, viral infections, and other inflammatory disorders. Furthermore, it has been proposed that A1 reactive astrocytes may have harmful consequences in neurodegenerative disorders such as ALS, AD, MS, PD and Huntington’s Disease (Liddelow and Barres, 2017). A2 astrocytes despite being considered as protective, in neuropathic pain and epilepsy may be the cause of aberrant synapses (Eroglu and Barres, 2010).

Microglia and astrocytes have shown bidirectional communication during neuroinflammation processes in CNS. When the brain parenchyma is insulted, microglia are the first to respond, converting the signals into various chemicals through known signaling pathways such as nuclear factor kappa B (NF-κB) or mitogen-activated protein kinase (MAPK). These microglia-derived molecules such as IL-1β or TNF-*α* have the ability to control astrocytes’ stimulus-dependent responsiveness. Furthermore, it has been demonstrated that limiting microglial inflammatory signaling in animal PD model, reduces astrocytic neurotoxicity and as a result neurodegeneration (Yun et al., 2018; Bhusal et al., 2023). Moreover, astrocytes, if activated, can also regulate the morphologies of microglia. Release of for example IL-1β, IL-10, CCL2 or TNF-α is described as being able to modulate microglia polarization (Wang et al., 2021). According to recent data, these glial cells’ interactions are crucial for either speeding up or slowing down the development of neuroinflammation and the disease as a whole (Doroszkiewicz et al., 2021; Hochuli et al., 2024; Mahbub et al., 2024).

In addition to the traditional innate immune cells such as microglia and astrocytes, oligodendrocytes support innate immune responses as well by generating immunomodulatory cytokines and chemokines. Oligodendrocytes can not only induce protective and regenerative processes during CNS traumas and diseases, but unfortunately they can potentially exacerbate neurodegeneration via inadequate myelin synthesis or repair (Stephenson et al., 2018). In many CNS illnesses, the interaction between oligodendrocytes and microglia is of great interest.

It has long been believed that NK cells are lymphocytes that destroy cancerous and virally-infected cells. Nonetheless, T-cell responses and homeostasis have been found to play regulatory roles in recent investigations. Reduced numbers of NK cells have been identified in depression, and dysfunction of these regulatory activities has been associated with MS (Suzuki et al., 2017). Mast cells may be harmfully responsible for maintaining CNS inflammation because they mediate BBB permeability and immune cell recruitment into the brain (Skaper et al., 2017). According to recent research, mast cells interact with gut permeability and microbiota. As a result, they may have an impact on a variety of disorders where the gut microbiota plays a significant role, such as epilepsy, ALS, MS, PD (Girolamo et al., 2017).

### 2.2 Adaptive responses in neuroinflammation

Changes in T- and B-cell subsets and (auto)antibody levels in the blood, cerebrospinal fluid (CSF), and brain tissues during disease provide evidence for the role of adaptive immunity in neurodegenerative disorders (Cova et al., 2017). The majority of mononuclear cells in normal cerebral spinal fluid (70–80%) are CD4 + T cells with a central memory characteristic, though B lymphocytes and NK cells are present at lower frequencies than in blood (De Graaf et al., 2011; Lee Mosley, 2015).

For example, the profiles of cytokines and chemokines can be used to determine whether a certain cell subset plays an anti-inflammatory (Th2 or regulatory cells, Tregs) or harmful (Th1 or Th17) role. The range of autoimmune encephalopathy syndromes, which includes paraneoplastic neurological disorders (PNND), best exemplifies the function of adaptive immune responses in neurological diseases (Kalman, 2017; Balint et al., 2018). Treatment for many of these illnesses often consists of removing the tumor that expresses the abnormal antigen in PNND. Traditionally, MS has been defined by the invasion of the CNS by T and B lymphocytes. Although AD is predominantly caused by inflammation induced by CNS-resident microglia, there is known evidence of T-cell involvement (McManus et al., 2015). Adaptive immunity is also rapidly receiving attention in PD and ALS. For instance, higher blood Th17 cell counts are seen in early Parkinson’s disease, and some of these T-cells are known to identify *α*-synuclein (Sulzer et al., 2017). However, it is uncertain if these T-cells are harmful. During X-ALD, T- and B-cells are also visible in the CNS and could be a subsequent event (33). In a transgenic mouse model of AD, T-cells help remove plaques, but they also cause pathology and cognitive deficits that can be treated with IL-2 or anti-CD3 therapy (Alves et al., 2017; Laurent et al., 2017). Studies demonstrating that transplanting young mice’s splenocytes enhanced spatial learning and memory in amyloid precursor protein (APP)swe/PSENldE9 transgenic mice provide evidence that T-cells are more likely to be helpful in AD (Wang et al., 2015). It is unclear if older T-cells in AD are harmful, but early protection may be brought about by a reduction in the proliferation of hematopoietic stem cells, which is known to occur with age. This reduction can lead to a decline in memory and naïve B-cells, lower antibody levels, and a decrease in the quantity and function of T-cells, which are identified by an inverted CD4+/CD8 + ratio and an accumulation of CD8+/CD28 − cells (Olsson et al., 2001). Anti-inflammatory medications that alter the disease’s natural progression are effective proof that the adaptive immune response is important in the early stages of multiple sclerosis. Despite the widespread belief that the illness is caused by T cells, CD20 treatment shows unexpected efficacy, suggesting that B cells also play a crucial part in the illness. Recent data further supports this, showing that memory B-cells are important targets for immunotherapy that effectively treats relapsing–remitting multiple sclerosis (Baker et al., 2017). Approaches aimed at enhancing the adaptive immune response, however, are less successful when given to patients whose disease is progressing, suggesting that B-cells have a less significant role in the course of the illness.

Apart from the cellular component, immunoglobulins, such as oligoclonal IgG in the CSF, are a diagnostic marker for multiple sclerosis. Nevertheless, it is not yet known if these antibodies are harmful or only result from abnormal intrathecal B-cells stimulated by an Epstein–Barr virus infection which is discussed as being connected to the disease. This is still a contentious issue that needs more research. The closely similar disorder NMO (neuromyelitis optica), which was formerly included in the MS spectrum, is now recognized as a distinct entity due to the discovery that the target antigen of the antibody is the water channel anti-Aquaporin 4 (AQP4). This stands in contrast to the role that antibodies may play in MS. It has been shown *in vitro* and in animal models that antibodies to AQP4 are pathogenic, causing damage to astrocytes (Vaknin-Dembinsky et al., 2014; Stephenson et al., 2018b).

### 2.3 Counteractive mechanisms of neuroinflammation

**Figure 1:**
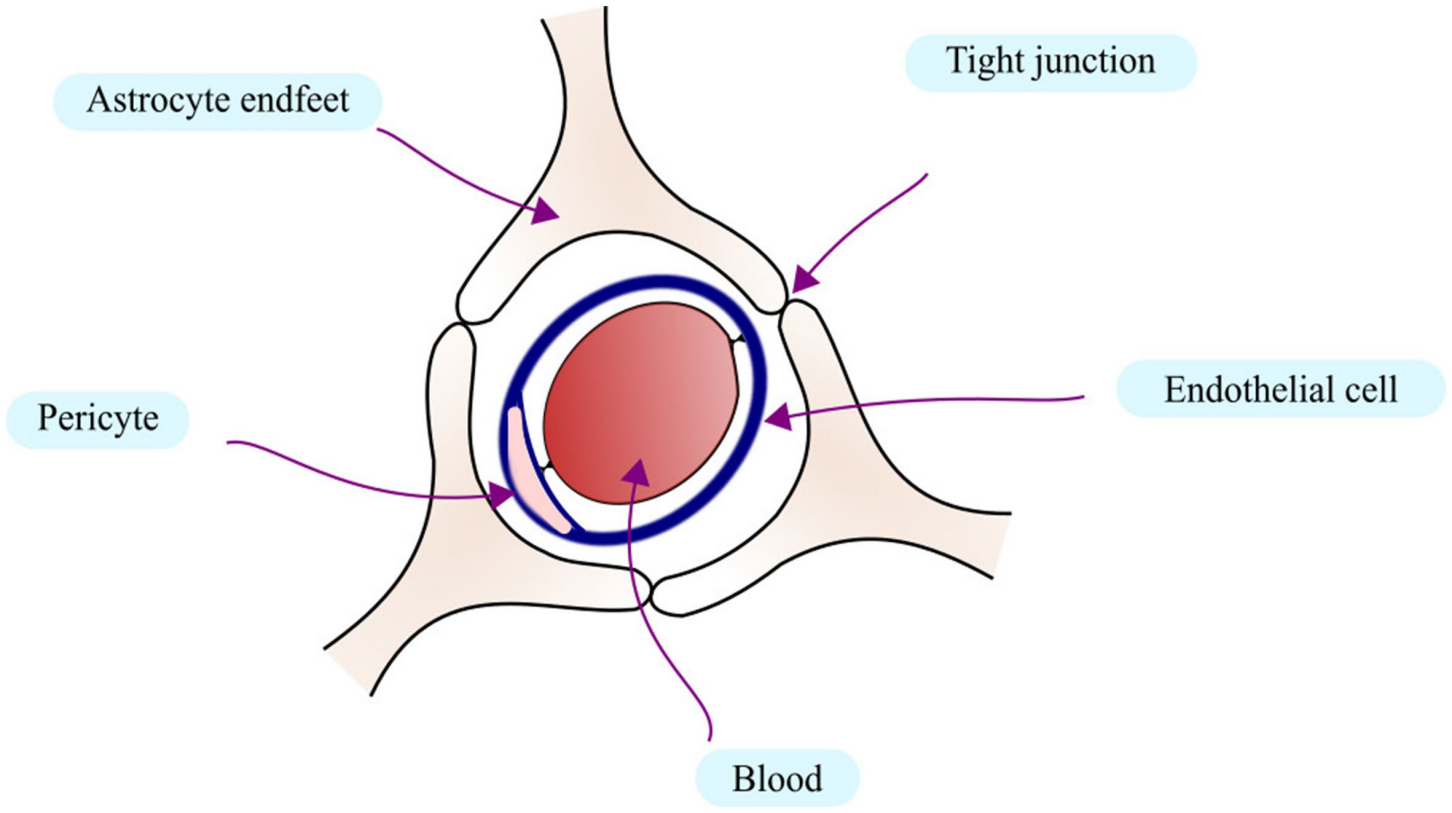
The schematic presentation of the Blood Brain Barrier.

The central nervous system has evolved defense mechanisms to restrict both the infiltration of immune components and the formation of immunological activation within the tissue. In the middle of the 20th century, the so-called “immune privilege” phenomena was identified. The blood–brain barrier, which is intended to restrict the entry of solutes and ions into the CNS, has a role in limiting immune privilege in the brain (Carson et al., 2006). The capillary venules facilitate the selective entrance and exclusion of substances from the central nervous system. On the other hand, cell migration occurs at the post-capillary venules, where it is regulated by adhesion molecules, cytokines, and chemokines, as well as their receptors (Owens et al., 2008). The BBB is presented in Figure 1.

The suppressive environment within the CNS not only controls the physical characteristics of the BBB but also potentially harmful immune responses. Another barrier is blood–cerebrospinal fluid barrier (BCSFB), which is formed by the epithelial cells of the choroid plexus. Its epithelial cells regulate the release of CSF into the ventricular brain system (Brown et al., 2004). On the other hand, secretion across the BBB’s capillary endothelium is the primary source of the interstitial fluid (ISF), which comprises the remaining extracellular fluid in the brain (Abbott, 2004). ISF freely exchanges information with CSF across multiple places, and its percentage of CSF is thought to be between 10 and 60% (Abbott et al., 2010).

Microglia and astrocytes both have a significant part in this control, but neurons are thought to be primarily passive and only the targets of immunological responses. Early in brain development, microglia enter the brain and assume a “protective” role while at resting state. They disperse evenly throughout the central nervous system and create a network of possible effector cells. Microglia in their activated state and protective phenotype play the opposite role to peripheral macrophagesby reducing inflammation (Wang et al., 2023). Additionally, astrocytes—the first cells that immune cells entering the central nervous system encounter—play this role. Astrocytes have a wide range of molecular pathways to cause activated T cells to undergo apoptosis. They also suppress the activation of T helper 1 (Th1) and T helper 2 (Th2) cells, as well as the proliferation and effector activities of activated T cells (Amor et al., 2010).

Many of the products of neurons, such as neuropeptides and transmitters, along with the neuronal membrane proteins CD22, CD47, CD200, CX3CL1 (fractalkine), intercellular adhesion molecule (ICAM)-5, neural cell adhesion molecule (NCAM), semaphorins, and C-type lectins, all regulate inflammation, refuting the notion that neurons only have a passive role (Tian et al., 2009). Additionally, through the Fas–Fas ligand pathway (CD95–CD95L), neurons actively promote T-cell death and display low amounts of major histocompatibility complex (MHC) molecules. Inhibiting inflammation is also linked to the neural expression of the cannabinoid (CB1) receptor (Barrie and Manolios, 2017). The autoimmune model of multiple sclerosis, known as experimental autoimmune encephalomyelitis (EAE), is more common in CB1 mutant mice. Additionally, neurons promote T-regulatory cell development by creating a local microenvironment where transforming growth factor-*β*1 (TGF-β1) predominates. However, damaged neurons are less able to withstand such assaults and therefore remain vulnerable. In short, the brain and spinal cord lose their immune-privilege status once they are sensitized to antigens in the central nervous system, even when every attempt is made to inhibit these reactions (Amor et al., 2010).

## 3 Neuroinflammation in neurodegenerative disorders

### 3.1 Alzheimer’s disease

Alzheimer’s disease is the most common neurodegenerative disorder that causes memory loss and cognitive impairment. The extracellular buildup of amyloid-beta (Aβ) plaque and intracellular neurofibrillary tangles (NFTs) are the pathological features of the illness. The hyperphosphorylated microtubule-binding protein tau, which controls the cytoskeleton range, makes up NFTs. The gamma (*γ*) and beta (β)-secretase enzymes catalytically cleave the amyloid precursor protein (APP) to produce Aβ oligomer and fibrils, which make up an Aβ plaque. Rare familial AD, consisting of about 5% of the cases, is caused by mutations in APP and γ secretase, indicating that Aβ may play a role in the pathophysiology of AD (Bertram et al., 2010; Murphy and Levine, 2010). Although the harmful significance of Aβ is established, the pathogenesis mechanism is still unclear. Aβ aggregates impact the immune system cellular and molecular components as well as neural development. Interestingly, it is widely recognized that glial cells, astrocytes, and microglia, can spread Aβ toxicity. As a kind of guardian, microglia monitor and identify alterations in the brain. Microglial activation caused by Aβ aggregates results in the release of pro-inflammatory cytokines, chemokines, reactive oxygen species (ROS), and nitric oxide (NO), which may be a factor in neuronal death (Akiyama et al., 2000; Kitazawa et al., 2004). Cellular receptors, including Toll-like receptors (TLRs) and receptors for advanced glycoxidation end-products (RAGE) on microglia, are contacted by Aβ aggregates. Genes involved in the downstream inflammatory response are transcriptionally activated as a result of this interaction (Reed-Geaghan et al., 2009). Considering astrocytes, Aβ aggregates have also been shown to bind to TLRs and RAGE. Because downstream target genes are induced, reactive astrocyte activation results in the production of harmful molecules (Paudel et al., 2020). When inflammatory reactions create loops with the creation of Aβ, they become harmful because they override the typical resolution mechanism of Aβ aggregates (Akiyama et al., 2000). The immunological cross-talk between microglia, astrocytes, and neurons is also unbalanced by the uncontrolled inflammatory response, which impacts the regulatory systems governing synaptic plasticity, neuronal survival, and cognition (Chun et al., 2018; Singh, 2022).

Studies, both preclinical and clinical, have demonstrated the critical role activated microglia play in the development of AD (Weitz and Town, 2012; Mirarchi et al., 2024). In the brains of AD patients, researchers have discovered microglia linked to Aβ plaques (Wisniewski et al., 1989, 1992; Akiyama et al., 2000). High immunoreactivity has been seen in microglia surrounding Aβ plaque toward chemokine receptors (CCR3, CCR5), cytokines (IL-1, MCP-1, MIP-1α, IL-1β, TNFα, and IL-6), and activation markers (MHCII and COX2) (Akiyama et al., 2000). It has been found that MCP-1 induces astrocyte chemotaxis and recruitment around Aβ plaque (Wyss-Coray et al., 2003). Furthermore, Aβ-induced activation of microglia results in the production of ROS via the NADPH oxidase system and RNS through the regulation of inducible nitric oxide synthase. These harmful elements promote neurotoxicity, impede Aβ clearance, and increase amyloidogenesis (Rojo et al., 2008). Conversely, research results have demonstrated that activated microglia have also a neuroprotective impact. According to Shaftel et al., overexpressing IL-1β in the APP/PS1 mice model lowers the amount of Aβ plaque via boosting the quantity of anti-inflammatory activated microglia (Shaftel et al., 2007). By promoting gliosis and microglial activation, IL-6 overexpression in APP transgenic mice has been demonstrated in another work to be beneficial against Aβ plaque accumulation (Chakrabarty et al., 2010). Consequently, conflicting research indicates that inflammation mediated by microglia may be either neurotoxic or neuroprotective. The magnitude, duration, and timing of microglia activation could all influence the course of disease (Singh, 2022).

Not only microglia but also astrocytes contribute to the neuroinflammation. In order to be activated they have undergone morphological and functional alterations due to pathological conditions. These astrocytes are characterized by hypertrophy of the cell and excessive release of neurotoxic chemicals. Prominent indicators like vimentin, which causes cell enlargement, and glial fibrillary acidic protein (GFAP) are expressed more often in reactive astrocytes. Activated astrocytic cells have been observed in close proximity to Aβ plaque in the context of AD, as demonstrated by data from post-mortem and rodent model investigations (Kato et al., 1998; Wegiel et al., 2001). In the APP/PS1 animal model of AD, the reduction of GFAP and vimentin raises the Aβ plaque load and decreases the astrocytic reactivity (Kraft et al., 2013). Additionally, there also might occur a neuroprotective mechanism for reactive astrocytes. In order to minimize collateral damage caused by neurotoxic Aβ species, it encircles (forming a physical barrier termed glial scar) and infiltrates Aβ plaque. Moreover, research conducted *in situ* and *in vitro* has demonstrated that reactive astrocytes use phagocytosis to eliminate or lessen Aβ deposits (Wyss-Coray et al., 2003; Xiao et al., 2014). Furthermore, as the study by Gomez-Arboledas et al. suggests, impairment in an inflammatory response, a neuronal circuit, and Aβ-mediated disease may be reversed by enhancing the phagocytic capability of astrocytes (Gomez-Arboledas et al., 2018).

The diagnosis of AD is generally performed through a series of neuropsychological tests, Mini-Mental State Examination (MMSE), laboratory tests such as assessing the concentrations of Aβ42, Aβ42/40 ratio, total Tau and pTau181 proteins in the CSF and brain imaging tests (Mayo Clinic, 2024; Porsteinsson et al., 2021). Establishing new biomarkers which will help to discover disease in the early stages and then help in possible treatment discovery is needed. Therefore, understanding which proteins are secreted in the neuroinflammation might be helpful. Activated microglia and leukocytes release interleukins which stimulate neutrophil and macrophage activity, but also encourage T-lymphocyte-mediated toxicity. Pro-inflammatory cytokines include IL-1β, IL-6, IL-18, and tumor necrosis factor; chemokines like C-C motif chemokine ligand 1 (CCL1), CCL5, and C-X-C motif chemokine ligand 1 (CXCL1); small-molecule messengers like prostaglandins and nitric oxide; reactive oxygen species produced by innate immune cells in the central nervous system are indicative of neuroinflammation (DiSabato et al., 2016; Leng and Edison, 2021). Many of them were analyzed in the CSF and blood of patients with AD. Overall, compared to healthy controls, patients with AD had higher levels of IL-1β, IL-6, IL-12, IL-18, TNF*γ*, and TGFβ in both fluids; however, there are significant differences between the studies, raising doubts about the usefulness of these well-established inflammatory markers (Swardfager et al., 2010). Moreover, systemic inflammation affects these cytokine concentrations, which further complicates the interpretation of the data (Leng and Edison, 2021).

However, also anti-inflammatory proteins are broadly discussed during the course of the disease. IL-4, IL-10, and TGF-β are anti-inflammatory cytokines that block the synthesis of proinflammatory cytokines and their effects. The brain inflammation in AD patients is being counteracted in part by the anti-inflammatory cytokine IL-4. According to research conducted *in vitro*, IL-4 promotes the production of CD36 and amyloid beta-degrading enzymes, which speeds up the clearance of microglia (Su et al., 2016). Conversely, *in vivo* administration of IL-4 resulted in a decrease in amyloid beta buildup and an enhancement of cognitive abilities in animal models of AD. Two SNPs of IL-4 (− 590 C/T and − 1098 T/G) in the promoter region are thought to affect the risk of AD (Zhang et al., 2011). Inhibitory effects of IL-4 include blocking and suppressing cytokines generated from monocytes as well as TNF*α*, IL-1, and IL-8. Inhibition of IFN-γ and reduction in NO and TNF-α concentrations are additional mechanisms contributing to neuroprotection (Thakur et al., 2023). Furthermore, IL-10 has a complex role in AD due to its anti-inflammatory properties in the central nervous system, as well as its role in apoptosis, cell survival, and neuroprotection. It has been shown that when IL-10 utterance was driven by an adeno-associated virus in the brains of AD models, it increased neurogenesis and cognition, two key symptoms of AD. More research is necessary in light of the fact that cognitive dysfunction has been shown to deteriorate in AD animal models and that adeno-associated virus-mediated IL-10 production speeds up the accumulation of amyloid beta (Porro et al., 2020). Thus, more research linking IL-10 polymorphisms to AD-related symptoms will contribute to our understanding of the protein’s function in AD. IL-10 mRNA is found in the frontal and parietal lobes and plays a crucial role in homeostasis and cell survival. By suppressing cytokine receptors like TNF-*α* and IL-1, and blocking receptor activation in the brain, IL-10 is believed to reduce inflammation. It interacts with cell surface receptors (IL-10Rs) on glial cells in the brain region (Rubio-Perez and Morillas-Ruiz, 2012; Thakur et al., 2023).

Another aspect worth of mentioning are the choline-containing phospholipids (CCPLs). They are a group of lipid molecules that contain choline as a component of their structure. Their main roles are taking part in cell membrane formation, neurotransmitter synthesis, and neurovascular health with visible connection to neuroinflammation (Tayebati and Amenta, 2013). Alzheimer’s disease and other neurodegenerative illnesses are linked to deficiencies in CCPLs. Preclinical research and a small number of clinical trials suggest possible advantages for CCPL supplementation. For example, *α*-GPC has been shown to boost acetylcholine release in the hippocampus, which is essential for memory and learning has shown cognitive gains in animal models (Traini et al., 2013). A paper by Roy et al. (2022) describes that CCPLs have anti-inflammatory and antioxidant effects. Therefore, deficiency of that molecule accelerates neurodegeneration and brain dysfunction in AD by increasing oxidative damage and inflammatory cytokine activity (Roy et al., 2022).

### 3.2 Parkinson’s disease

**Figure 2:**
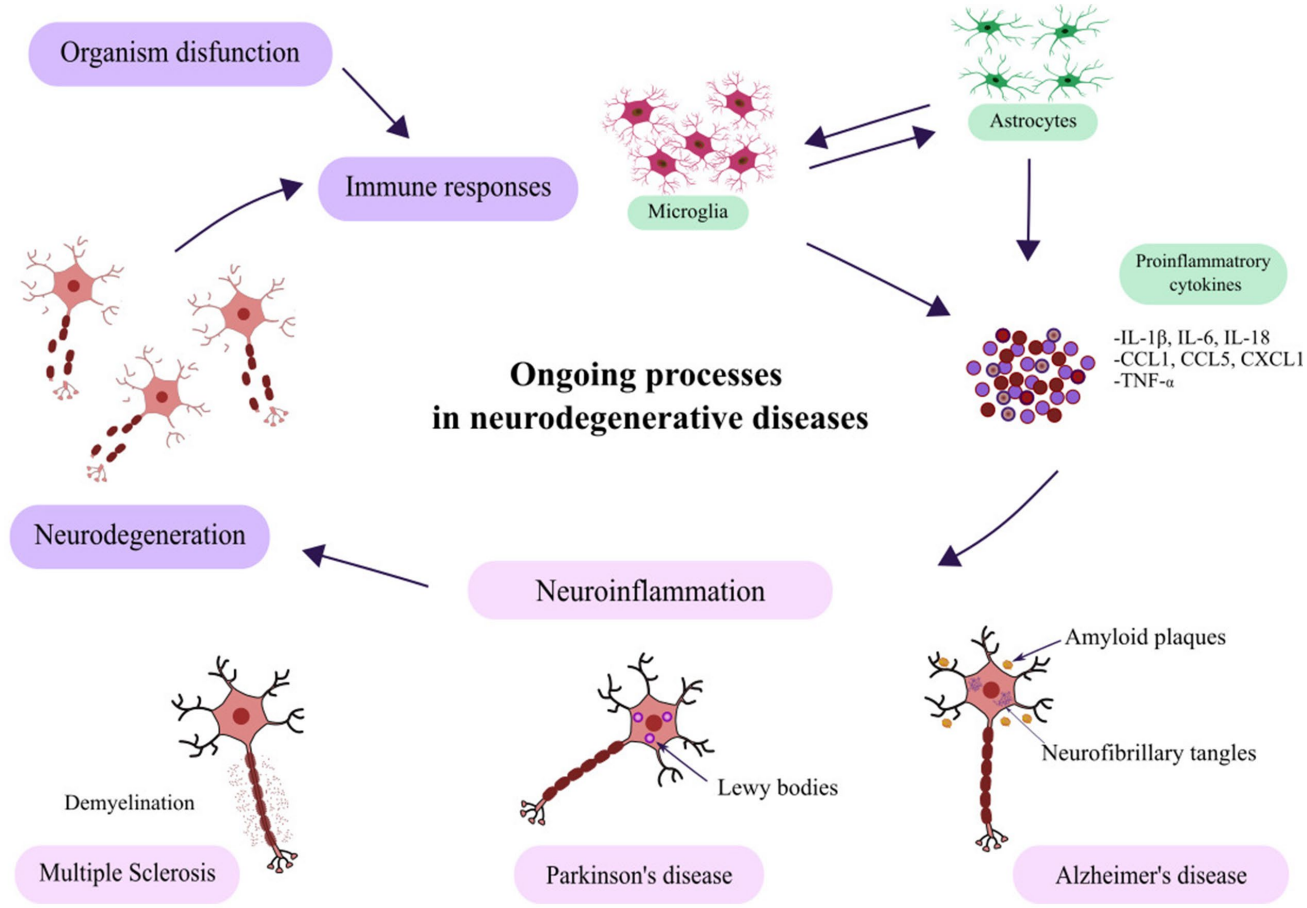
Schematic representation of ongoing cellular and molecular processes involved in neurodegenerative disorders, including protein aggregation, mitochondrial dysfunction, oxidative stress, neuroinflammation, and synaptic loss.

Parkinson’s disease is the most prevalent type of movement disturbance among the elderly (Kalia and Lang, 2015). Tremor, difficulty with movement, and issues with balance and coordination are common signs of PD. The pathogenic characteristic is the widespread aggregation of intracellular protein *α*-synuclein (α-syn) and the death of dopaminergic neurons in the brain’s substantia nigra pars compacta (SNpc) in the basal ganglia (Wang et al., 2018; Isik et al., 2023). Subsequent misfolded α-synuclein neurotoxicity can be further enhanced by neurotoxic cascades including genetic (Kim and Alcalay, 2017; Domingo and Klein, 2018), environmental (Ascherio and Schwarzschild, 2016), and immune factors (De Virgilio et al., 2016), leading to neurodegeneration in nearby brain areas. Numerous genetic variations have been linked to Parkinson’s disease by genome-wide association studies. Significant insights into the role of neuroinflammation in PD pathogenesis have also been gained from studies of animal models, neuroimages, and postmortem pathology (McGeer et al., 1988; Theodore et al., 2008). These researches suggest that cytokine-induced inflammatory responses may be crucial in the course of the disease (Liu et al., 2022). What is more, defective autophagy has been linked to neurodegenerative illnesses and may lead to the aggregation of α-syn (Spencer et al., 2009; Arotcarena et al., 2019). It’s possible that molecular processes linked to oxidative stress, inflammation, or autophagy promote one another as neurodegenerative diseases advance. Alpha-syn aggregation and mitochondrial dysfunction are both seen in PD-damaged neurons. It is believed that these pathogenic alterations intensify one another (Poewe et al., 2017). Research has shown that oxidative stress, which is a major factor in cell death brought on by a variety of pathophysiological circumstances, is a direct result of mitochondrial failure. Oxidative stress occurs when a cell produces more ROS than antioxidants or radical scavengers can readily absorb. ROS generation is significantly influenced by mitochondria (Cui et al., 2012). Oxidative stress has been demonstrated to rise in the brain tissues of Parkinson’s disease patients. On the other hand, the precise point in the illness at which oxidative stress takes place is unknown. PD is associated with a number of possible pathogenetic pathways, including increased oxidative stress and mitochondrial dysfunction, which can result in the loss of lysosomes (Dehay et al., 2010; Guzman et al., 2010) and also are linked to genes (Siddiqui et al., 2012). SNpc neuron loss may also result from oxidative stress-induced autophagy (Janda et al., 2012). Chronic inflammation, primarily caused by microglial cells, can cause oxidative stress and mitochondrial malfunction, which can lead to *α*-syn aggregation and neuropathological processes that ultimately cause dopaminergic neuronal degeneration in Parkinson’s disease (He et al., 2020). It is important to recognize that Parkinson’s disease is a multi-systemic illness that affects peripheral immune cells, including T cells and monocytes from the innate and adaptive immune systems, respectively (Harms et al., 2021; Isik et al., 2023; Harms et al., 2021). Depending on which cell types from the innate and adaptive immune systems are involved, acute and chronic responses are typically differentiated. Triggering factors influence each other when they result in activated microglia and/or malfunctioning neurons. The presentation of antigen by microglia then stimulates T cells, which in return triggers B cells to generate antibodies. The adaptive immune system eventually produces a persistent inflammatory response and destroys neurons in Parkinson’s disease when this cycle continues (Kannarkat et al., 2013). Reactive state microglia, a key participant in neuroinflammation, is also in charge of astrocyte activation. Maintaining the integrity and permeability of the blood–brain barrier is one of the many functions of astrocytes. By secreting neurotrophic factors, such as nerve growth factor (NGF), brain-derived neurotrophic factor (BDNF), cerebral dopamine neurotrophic factor (CDNF) and glial cell line-derived neurotrophic factor (GDNF), they support neuron plasticity, survival, development, protection, and tissue restoration. As previously indicated, these factors build the glial scar for the regeneration of neural tissue and combat ROS by producing antioxidants (Miyazaki and Asanuma, 2020; Heithoff et al., 2021). Together, astrocytes and microglia can also remove extracellular *α*-syn, protecting neurons in the process (Lee et al., 2008; Hua et al., 2019). Astrocytes have both pro- and anti-inflammatory characteristics, similar to microglia. They have the ability to control microglia activity when there is inflammation (Liddelow and Barres, 2017). T cells have been reported to be higher in the SN of PD patients (Brochard et al., 2009). Furthermore, research has demonstrated that the pathogenicity of Parkinson’s disease is also influenced by humoral adaptive immunity (Yanamandra et al., 2011). When combined, these findings suggest that both the innate and adaptive immune systems are activated in PD (Isik et al., 2023). The processes ongoing during neurodegeneration in selected diseases are presented in the Figure 2.

As previously described, a major factor in the pathophysiology of Parkinson’s disease is the aggregation of the aberrant, insoluble form of *α*-synuclein. Misfolded α-synuclein plays a role in the dysregulation of microglial TLR2 or TLR4-mediated signaling pathway mediated by PAMPs or DAMPs. This pathway ultimately activates NFκB and myeloid differentiation primary response 88 (MyD88), which in turn causes the production of TNF-α and IL-1β (Béraud and Maguire-Zeiss, 2012). Aggregated α-synuclein treatment of BV2 murine microglial cells or primary microglia increases the production of TNF-α, IL-1β, interferon (IFN)-*γ*, and monocyte chemoattractant protein (MCP)-1 (Reynolds et al., 2008; Lee et al., 2010; Couch et al., 2011). A study by Panicker et al. showed that aggregated α-synuclein binds to TLR2 and CD36, which are receptors on the microglial surface cell membrane. Fyn kinase is then recruited, activating and phosphorylating protein kinase C-delta (PKCδ). This increases PKCδ-dependent activation of the NFκB pathway, which in turn increases the production of IL-1β (Panicker et al., 2019). In mouse microglia, TLR2 knockout decreases α-synuclein uptake (Kim et al., 2013). α-synuclein is incorporated into autophagosomes through the activation of the TLR-4–NFκB pathway (Choi et al., 2020b; Choi et al., 2020a). In BV2 mouse microglia or TLR4-knockout primary mouse microglia, functional inhibition of TLR4 inhibits α-synuclein uptake and stops TNF-α and IL-6 production (Fellner et al., 2013). α-Synuclein also raises IFN-γ expression in microglia, which causes neuronal MHC-I expression. As a result, CD8 + T lymphocytes can target neurons specifically (Cebrián et al., 2014; Liu et al., 2022; Cebrián et al., 2014).

Since clinical signs of Parkinson’s disease are typically seen in severe stages of the disease, early treatment intervention is not currently available. However, the diagnosis of PD is still mostly based on clinical symptoms. Early PD detection, tracking the course of the disease, and evaluating the effectiveness of treatment all depend on biomarkers. α-synuclein, tau, and Aβ42 in CSF, blood, and other bodily fluids have garnered significant attention from researchers as potential molecular biomarkers of Parkinson’s disease (Parnetti et al., 2013, 2014; Hall et al., 2015; Mollenhauer et al., 2017). Potential biomarkers for the neuroinflammatory etiology of PD may include substances that take part in neuroinflammation and are released during the course of the disease (Reale et al., 2009; Parnetti et al., 2013; Qin et al., 2016). Since it is difficult to acquire living human neurons from PD patients, the CSF serves as a suitable substitute and can be applied to identify molecular alterations that underlie the neurodegenerative pathogenesis. Peripheral blood may additionally indicate the release of inflammatory chemicals from areas of the brain that have declined. The peripheral involvement of PD pathogenesis is further indicated by changes in inflammatory biomarkers in the blood of PD patients. A study by Liu et al. summarized assessing these potential neuroinflammatory proteins in PD patients (Liu et al., 2022). They described results from recent research which has identified peripheral biomarkers such as TNF-α/soluble TNF-receptors (sTNFRs), IL-1β, IL-2, IL-6, IL-10, high-sensitivity C-reactive protein (hsCRP), and regulated upon activation, normal T cell produced and presumably released (RANTES) being involved in PD neuroinflammation (Liu et al., 2022).

### 3.3 Multiple sclerosis

Multiple Sclerosis is an inflammatory disease of the CNS. This chronic illness results in multifocal lesions with confluent foci of myelin loss and comparatively preserved axons (Lassmann, 2018; Reich et al., 2018). Although the underlying causes of inflammatory demyelinating lesions are still unknown, the general consensus is that an autoimmune mechanism is to blame. In contrast to AD and PD, there is no clear familial form of MS that could help to identify endogenous starters of disease. Once established, acute or active MS lesions might become shadow plaques with remyelinating patches or chronic inactive plaques with glial scars. Anywhere in the CNS, including the gray and white matter regions, might have lesions. The optic nerve, brain stem, spinal cord, periventricular white matter, and the gray matter next to the subarachnoid space are sites of predilection. Demyelination surrounded by a central vein, which appears to be the source of the inflammatory response and the point from which the demyelinating injury appears to spread throughout the brain, constitutes a pathological feature of white matter lesions. Lesions in the cortex can be perivascular, but there are also many demyelinated sites that lack a central vein and are linked to leptomeningeal inflammation. These areas are referred to as subpial lesions. The inflammatory cells that are largely located in the perivascular or meningeal area and infiltrate active MS lesions are mostly composed of CD8 + T-cells, with a minor contribution from CD4 + T-cells and B-cells (Dendrou et al., 2015). Myeloid cells, which include brain-resident activated microglia and monocyte-derived macrophages that have migrated into the brain, make up the largest percentage of immune cells in active MS lesions (Bogie et al., 2014; Mishra and Wee Yong, 2016; Cantuti-Castelvetri et al., 2022). Demyelinating processes are hypothesized to be induced by myeloid cells in conjunction with soluble substances released by lymphocytes (Lassmann, 2018). As a result, many immunosuppressive or immunomodulatory drugs that target this auto-inflammatory response have shown promise in the management of multiple sclerosis by lowering the development of new lesions (Montalban et al., 2018; Faissner et al., 2019). Although the disease is primarily driven by auto-inflammation, which results in recurrent episodes of demyelination, the ensuing lesions will eventually cause immune responses that are injury-induced and counter-reactive. When tissue damage occurs, the immune system is activated and starts coordinating regeneration processes to try and bring the body back to equilibrium. When compounds that are typically enclosed within cells become exposed to the external environment, they come into contact with pattern recognition receptors, which in return causes reparative inflammation (Karin and Nature, 2016; Medzhitov, 2021). In order to obtain clearance of the distressing stimuli, signaling downstream of pattern recognition receptors starts an inflammatory response (Takeuchi and Akira, 2010; Cantuti-Castelvetri et al., 2022).

Magnetic resonance imaging (MRI) is currently the most dependable and frequently used diagnostic method for multiple sclerosis. More specifically, white matter and gray matter MS lesions are distinguished using T2-weighted MRI scans. These lesions show mixed pathology of demyelination and neuro-axonal destruction along with inflammation (Zivadinov et al., 2018). Another part of the diagnosis is analyzing the CSF of potential MS patients. Significantly higher levels of CSF neurofilament light chain (cNfL) and immunoglobulin (Ig)G-index were observed in certain patients whose condition was determined to be inactive by clinical scales and/or MRI (Sellebjerg et al., 2017). The ratio of IgG to albumin in the CSF to that in the serum is known as the IgG index (Villar et al., 2008). Evaluation of the immunoglobulins and oligoclonal bands are two indicators of inflammation that can be found in the CSF and are useful for diagnosing conditions, but they are not the best biomarkers for predicting relapse and progression. Since it can be difficult to identify the number of bands present, measuring oligoclonal bands is not highly sensitive. They are also not very selective because increased oligoclonal bands can be caused by anything that causes persistent inflammation (Becker et al., 2015). Numerous assays have been developed to measure NfL levels over the last thirty years. Cytoskeletal proteins called neurofilaments are discharged into the CSF and bloodstream from injured axons. Research has also revealed a correlation between higher cNfL levels and more CD4 + T cells, which have been linked to the inflammation observed in MS and the development of RRMS into SPMS (Sospedra and Martin, 2005; Salzer et al., 2010). According to preliminary research, MS patients’ cNfL levels are higher during acute and active relapses than in healthy controls (Malmeström et al., 2003). In MS patients, there is a positive connection between serum NfL (sNfL) and cNfL (Gisslén et al., 2016; Disanto et al., 2017). Before treatment, sNfL levels were generally higher in MS patients than in healthy controls. What is more, sNfL levels decreased after disease-modifying therapy (Disanto et al., 2017). It has also been demonstrated that more efficacious treatments lower NfL levels more successfully than conventional ones. Furthermore, T2 lesion volumes have been linked to sNfL levels (Cantó et al., 2019). According to certain findings, there is a significant association between the number of active lesions visible on MRI scans and sNFL levels (Akgün et al., 2019; Uher et al., 2020; Yang et al., 2022).

Although in MS inflammation in the CNS is caused by primarily an autoimmune response, the proinflammatory proteins could also be analyzed in affected patients. T helper (Th) 1 and Th17 cells, which are pro-inflammatory, generate cytokines including interleukin (IL)-17, interferon (IFN)-*γ*, and TNF-*α*, whereas regulatory T (Treg) and Th2 cells, which are anti-inflammatory, produce IL-10 and IL-4. As evidenced by research in pediatric MS where serum levels of the anti-inflammatory cytokine IL-10 were predictive of relapse compared to other pro- and anti-inflammatory cytokines, measuring these cytokines and cellular alterations can reflect the kind of disease (Cala et al., 2016). Consequently, immunological signatures can be utilized in conjunction with the aforementioned markers as a composite to further distinguish between the pathology and activity of the underlying disease. Furthermore, it has been discovered that CXCL 13—a chemokine ligand with a C-X-C motif—is associated with a worsened prognosis, RRMS exacerbations, and the transformation of CIS into MS. CXCL13, however, is non-specific because individuals with illnesses also had elevated levels (Khademi et al., 2011). According to one study, CSF and plasma levels of eotaxin-1 (CCL11), particularly in SPMS patients, were linked to the length of the disease. Additionally, they discovered that CSF levels of IL-12B, MIP-1a, CXCL9, cluster of differentiation (CD)5, and MIP-1a, as well as plasma levels of oncostatin and hepatocyte growth factor, were linked to MS (Huang et al., 2020). They also discovered that C-C motif chemokine ligand (CCL) 20 was associated with the severity of the disease (Yang et al., 2022).

However, MS is described as an autoimmune disease activated microglia and astrocytes are also involved in the course of the disease. In the MS brain, macrophages and microglia exhibit a phenotype that lies in the middle of pro- and anti-inflammatory (M1) and microglia (M2) cells (Vogel et al., 2013). Nevertheless, they strongly express nicotinamide adenine dinucleotide phosphate (NADPH) oxidase at regions of active demyelination and tissue destruction, suggesting oxidative tissue damage (Fischer et al., 2012; Lassmann, 2018). It is becoming more well-acknowledged that astrocytes play a crucial role in the emergence of MS lesions. Astrocytes were formerly thought to only respond by creating a glial scar at a later, post-inflammatory stage, but they are now recognized as early and active participants in lesion pathogenesis (Brosnan and Raine, 2013; Ponath et al., 2017). Astrocytes take on a hypertrophic morphology in active lesions, which is typified by a substantial expansion of the cell soma and a decrease in process density (Brosnan and Raine, 2013). Prominent astroglial hypertrophy is usually suggestive of significant tissue damage and may be brought on by oligodendrocyte loss in MS lesions, which in turn disrupts astrocyte–oligodendrocyte networks (Orthmann-Murphy et al., 2008; Brosnan and Raine, 2013). Furthermore, hypertrophic astrocytes may experience significant harm that results in the glia limitans retracting or losing their position in the basal lamina surrounding blood vessels, so potentially facilitating immune cells’ access to the central nervous system (Brosnan and Raine, 2013; Ponath et al., 2018). Reactive astrocytes may reduce inflammation, support neuroprotection, and aid in lesion healing even though they additionally induce inflammatory and neurotoxic reactions in MS lesions. BDNF is a molecule that is produced by neurons and astrocytes in the healthy central nervous system and has CNS-trophic effects (Huang and Reichardt, 2001; Lee et al., 2012). A more severe clinical course with greater axonal loss was seen in EAE patients with astrocyte-specific deletion of BDNF (Linker et al., 2010). Furthermore, increased remyelination was seen in the cuprizone mouse model as a result of increased BDNF synthesis by astrocytes brought on by metabotropic glutamate receptor stimulation (Fulmer et al., 2014). Nonetheless, an independent investigation revealed that astrocytes produce nitric oxide as a result of signaling via the BDNF receptor TrkB. When EAE emerged in mice with a genetic deletion of TrkB specific to astrocytes, the disease severity declined, and astrocytic and lesional iNOS expression was also decreased (Colombo et al., 2012). According to these findings, BDNF produced by astrocytes increases the synthesis and release of toxic NO in astrocytes as well as other cell types, eliciting neuroprotective effects. Reactive astrocytes and immune cells in MS lesions are the main sources of BDNF, and the lesion’s immediate area of reactive astrocytes and neurons showed a substantial upregulation of the BDNF receptor TrkB. This implies that BDNF may have both a degenerative and protective function (Stadelmann et al., 2002; Ponath et al., 2018).

## 4 Similarities and differences between diseases

### 4.1 Similarities between inflammation in AD, PD, and MS

Despite having different causes, AD, PD, and MS all share important neuroinflammatory pathways that lead to neurodegeneration. Microglial activation will come first. Chronic activation of microglia causes them to change from a neuroprotective to a neurotoxic state in all three disorders. Pro-inflammatory cytokines such as IL-1β, TNF-*α*, IL-6, and reactive oxygen species (ROS) are excessively released as a result of this activation, exacerbating neuronal injury (Akiyama et al., 2000; Cala et al., 2016; Liu et al., 2022). The microglia respond to tau tangles and Aβ plaques in AD, to α-synuclein aggregation in PD, and to demyelination and lesion development in MS which are the main causes underlying these diseases.

Glial scar development and astrocyte reactivity will be the next factors to be considered. Through the formation of glial scars and the release of inflammatory mediators, reactive astrocytes are involved in neuroinflammation in all three disorders. Even yet, the scars will have distinct causes and appear in different places. In AD, astrocytes release inflammatory cytokines and encircle Aβ plaques, which is typical. By intensifying oxidative stress, they aid in the death of dopaminergic neurons in the substantia nigra in Parkinson’s disease (Isik et al., 2023). Additionally, they hinder remyelination and healing in MS by forming glial scars in demyelinated lesions (Reich et al., 2018).

Furthermore, all three disorders share a common theme of mitochondrial malfunction and oxidative stress. Excessive oxidative stress causes apoptosis and damage to neurons. Increased production of ROS and nitric oxide (NO) by dysfunctional mitochondria contributes to inflammation and neurodegeneration. Oxidative stress is a contributing factor to synaptic impairment in AD. Dopaminergic neurons are more susceptible in Parkinson’s disease (PD) because of their elevated oxidative metabolism. Oxidative stress exacerbates demyelination and axonal damage in multiple sclerosis. Moreover, in all three circumstances, there are increased levels of IL-1β, IL-6, TNF-*α*, and IL-18 in the blood and cerebrospinal fluid (CSF). The BBB is disrupted by chronic inflammation, which makes it possible for immune cells and neurotoxic chemicals to enter the central nervous system. Especially in MS, BBB disintegration is very severe, which makes it easier for T-cells and B-cells to infiltrate.

Though more pronounced in MS, peripheral immune activation also plays a role in all three diseases. Via peripheral cytokine signaling and the gut-brain axis, systemic inflammation in AD and PD can worsen neurodegeneration. Lesion formation in multiple sclerosis is caused by the adaptive immune system’s (T-cells, B-cells) aggressive attack on myelin.

Additionally, AD, PD, and MS all exhibit the dual role of inflammation. At first, acute inflammation may be protective, assisting in the removal of debris and fostering healing. Nonetheless, in all three disorders, persistent inflammation causes synaptic dysfunction, neuronal loss, and the advancement of the disease.

The similarities are summarized in the Table 1.

**Table 1:** Compiled distinct and shared cellular and molecular mechanisms underlying Alzheimer’s disease (AD), Parkinson’s disease (PD), and Multiple Sclerosis (MS), summarizing pathological responses involving glial cells, oxidative stress, cytokine profiles, blood-brain barrier integrity, and peripheral immune contributions.

Mechanism	Alzheimer’s disease (AD)	Parkinson’s disease (PD)	Multiple sclerosis (MS)
Microglial activation	Response to Aβ and tau aggregates	Response to α-synuclein aggregates	Response to demyelination
Astrocyte reactivity	Neurotoxic gliosis	Enhances oxidative stress	Forms glial scars
Oxidative stress	Damages synapses	Selectively toxic to dopaminergic neurons	Exacerbates demyelination
Cytokine dysregulation	IL-1β, IL-6, TNF-α	IL-1β, IL-6, TNF-α	IL-1β, IL-6, TNF-α, IFN-γ
BBB dysfunction	Increases neurotoxic accumulation	Exacerbates dopaminergic loss	Enables immune cell infiltration
Peripheral immune involvement	Indirect through systemic inflammation	Indirect through gut-brain axis	Direct autoimmune attack

These shared neuroinflammatory processes highlight potential common therapeutic targets, such as modulating microglial activation, reducing oxidative stress, and preserving BBB integrity across AD, PD, and MS.

### 4.2 Differences between inflammation in AD, PD, and MS

While AD, PD, and MS share common neuroinflammatory mechanisms, they differ significantly in their underlying pathology, affected brain regions, primary symptoms, and immune involvement.

In these diseases there are different brain regions, that are affected. In AD, it begins in the hippocampus and cortex, which leads to memory loss and cognitive decline (Jaroudi et al., 2017). In PD substantia nigra with dopaminergic neurons are the most affected. This causes the motor symptoms of the disease (Isik et al., 2023). Additionally, primary targets in MS are white matter and oligodendrocytes in the CNS, which leads to demyelination and widespread lesions (Reich et al., 2018).

It is certain, that in all described diseases immune system is involved, however not in the same way. For example, neuroinflammation is AD is chronic with innate immune activation, while in MS adaptive immune responses are connected (T-cells and B-cells). In PD there innate immune response is also present, as in AD, but it affects mostly microglia. In MS there is a strong autoimmune involvement, while in AD and PD it is not present. Also, the involvement of peripheral immune system differs between diseases. In AD and PD systemic inflammation, gut and immune dysfunction are the main agents, whereas in MS immune cells directly attack myelin.

Also, there is definitely enormous difference between symptoms. Starting with cognitive impairment which is severe, connected to all parts of memory, language and problem-solving deficits in AD, which is only present in advanced stages of PD. In MS cognitive impairment might happen in some cases, but it is not always severe. In PD there are noticeable motor disfunctions such as tremors, rigidity and postural instability, which will not be present in AD. In MS they might demonstrate only with some kind of weakness, loss of coordination with present sensory symptoms such as numbness.

## 5 Conclusion

From the standpoint of inducers and effectors, every neurodegenerative illness can be identified by its unique way of triggering inflammatory reactions. Therefore, even while immune responses have helpful functions, they nonetheless need to be tightly controlled in order to avoid CNS damage. Immune cells experience withering as we age and activate repeatedly, suggesting that the central nervous system is not completely protected. One common factor in all cases of neuroinflammation is the activation of innate immune cells in the central nervous system, including astrocytes and microglia. While AD, PD, and MS share common neuroinflammatory mechanisms, they have distinct underlying causes, pathologies, and clinical manifestations. In AD accumulation of amyloid plaques and neurofibrillary tangles are the triggers of the neuroinflammation in PD it is connected to the alpha synuclein forming Lewy bodies that trigger the inflammatory response. In MS, the reaction is autoimmune in which the immune system mistakenly attacks its own myelin covering nerves, leading to inflammation and demyelination. TLRs and other pattern recognition receptors, in particular, that are expressed on microglia are probably essential for the initiation of inflammatory reactions, which are then further enhanced by astrocytes. Similarly, the generation of amplifiers and effector molecules, including cytokines (e.g., TNF-*α*, IL-1β, and IL-6), ROS, and NO, appears to be mediated via signal transduction pathways downstream of these receptors that control the activities of the transcription factors NF-κB and AP-1. For each of the neurodegenerative illnesses described in this review, a number of these variables may constitute universal neurotoxic factors. Although, MS differs from AD and PD in that it involves the adaptive immune system significantly. Nonetheless, further research regarding neuroinflammation should be conducted and discussed even more profusely to help in the diagnosis and potential treatment of neurodegenerative diseases. An interesting insight into the theme might be focusing on sequencing glial cells across the three diseases to compare their structure. To improve this area systems biology, neuroimaging, clinical phenotyping, and cross-disease treatment trials should all be integrated in a robust comparative, multidisciplinary, and time-resolved strategy. In order to create effective interventions for neurodegenerative and neuroinflammatory disorders, it may be possible to clarify if neuroinflammation is a cause, effect, or compensatory reaction.
